# Uso de una tira reactiva paraorina en la evaluación de las concentraciones de glucosa y bilirrubina en plasma en entornos con recursos limitados: un estudio de prueba de concepto

**DOI:** 10.1515/almed-2023-0153

**Published:** 2023-12-01

**Authors:** Laura Pighi, Davide Negrini, Brandon M. Henry, Gian Luca Salvagno, Giuseppe Lippi

**Affiliations:** Sección de bioquímica clínica y Facultad de Medicina, Universidad de Verona, Verona, Italia; Laboratorio clínico, División de nefrología e hipertensión, Hospital Pediátrico de, Cincinnati, OH, EE.UU.

**Keywords:** tira reactiva, orina, plasma

## Abstract

**Objetivos:**

El objetivo del presente estudio de prueba de concepto, era investigar si una tira reactiva comercial para orina aporta información potencialmente útil para la evaluación de glucosa y bilirrubina en muestras de plasma humano.

**Métodos:**

Se determinaron las concentraciones de glucosa y bilirrubina en 60 muestras anonimizadas de plasma residual de heparina de litio, en un analizador Roche COBAS 8000 y en la tira reactiva comercial para orina, tras pipetear 10 µL de plasma, en las almohadillas que ésta incorpora. Se llevó a cabo una comparación directa entre los resultados semicuantitativos de la tira reactiva y los de las muestras pareadas obtenidos con el analizador COBAS.

**Resultados:**

Se observaron leves diferencias entre el analizador COBAS y la tira reactiva en relación a la mediana de la concentración de glucosa (5,8 frente a 5,6 mmol/L, respectivamente; p=0,040). Por otro lado, no se observaron diferencias estadísticamente significativas en los valores de bilirrubina entre COBAS y la tira reactiva (11,2 frente a 8,6 μmol/L; p=0,090). El coeficiente de correlación de Spearman entre COBAS y la tira reactiva fue de 0,83 (IC 95 %, 0,73–0,90; p<0,001) para la glucosa en plasma, y 0,78 (IC 95 %, 0,66–0,87; p<0,001) para la bilirrubina en plasma. La concordancia acumulada entre COBAS y la tira reactiva fue alta tanto para la glucosa (88 %; coeficiente kappa, 0.75; IC 95 %, 0,58–0,92; p<0,001) como para la bilirrubina (88 %; coeficiente kappa, 0,76; IC 95 %, 0,60–0.92; p<0,001).

**Conclusiones:**

Los resultados del estudio de prueba de concepto indican que la tira reactiva comercial para orina de nuestro estudio posee un rendimiento aceptable en la determinación de las concentraciones de glucosa y bilirrubina en plasma, en comparación con las pruebas analíticas de referencia.

## Introducción

Una tira reactiva para orina es una herramienta sencilla de diagnóstico, ampliamente utilizada en contextos clínicos, que permite analizar rápidamente algunos de los componentes de la orina. Básicamente, está compuesta por una tira fina de plástico con diferentes cuadraditos o almohadillas que contienen reactivos específicos [1]. Cuando la tira se sumerge en una muestra de orina, las almohadillas pueden cambiar de color, lo que indica la presencia e incluso la concentración (semicuantitativa) de una sustancia concreta en la orina. Las tiras reactivas actuales se pueden leer visualmente, comparando los cambios de color de cada almohadilla con una escala o tabla de referencia situada dentro de la caja de la tira reactiva y proporcionada por el fabricante. O bien, se pueden leer automáticamente utilizando un moderno sistema de uroanálisis [2]. Cabe señalar que, aunque las tiras reactivas no permiten establecer un diagnóstico definitivo, son un método rápido y cómodo para el cribado de pacientes, especialmente en situaciones donde los recursos son limitados [3] o en áreas de difícil acceso (p.ej. catástrofes o desastres naturales) [4], donde puede que no se disponga de equipos de laboratorio y/o de dispositivos en el lugar de asistencia del paciente.

Aunque el empleo de tiras reactivas tradicionalmente se ha limitado al análisis de muestras de orina, existe la tentadora hipótesis de que se podrían utilizar para analizar suero o plasma, permitiendo así obtener de forma rápida y relativamente barata, información semicuantitativa sobre la concentración de algunos analitos en estas matrices biológicas, como glucosa, proteínas totales, urobilinógeno, bilirrubina, creatinina, pH, sangre, cetonas, nitritos, leucocitos y gravedad específica. Existen analitos, como la glucosa y la bilirrubina, cuyas concentraciones se pueden contrastar en orina y en plasma, lo que sugiere una utilidad diagnóstica en la evaluación de pacientes. El objetivo del presente estudio de prueba de concepto era investigar si una tira reactiva comercial para orina, proporciona información potencialmente útil a la hora de analizar la glucosa y la bilirrubina de muestras de plasma humano.

## Materiales y métodos

Seleccionamos aleatoriamente 60 muestras anonimizadas sobrantes de heparina de litio (3.5 mL tubo de sangre con heparina de litio; Vacutest Kima, Padua, Italia) obtenidas por la mañana en un día laboral de trabajo, en el Servicio de Medicina de Laboratorio del Hospital Universitario de Verona y tras realizar un perfil bioquímico clínico (que incluía glucosa, proteína, bilirrubina y creatinina). Las muestras fueron procesadas en un analizador Roche Cobas 8000 (Roche Diagnostics, Basel, Suiza). La identidad de las muestras se sustituyó por nuevos identificadores, para impedir el reconocimiento de las mismas, pero conservando su idoneidad para el procesamiento y análisis de datos. Inmediatamente después del procesamiento de las muestras en el analizador Roche, se vertieron 10 µL de plasma con una micropipeta en cada una de las diez almohadillas de una tira reactiva comercial para orina (AUTION Sticks, Arkray, Kyoto, Japón), cuyas características han sido ampliamente descritas en otras publicaciones [5]. Tras 10 segundos, se retiró el plasma sobrante, colocando la tira en un trozo de papel de seda, y se interpretaron visualmente los resultados de la tira en los siguientes 60 segundos, comparando el color de la almohadilla con el color indicado en la tabla del frasco. Dos miembros experimentados del laboratorio interpretaron todas las lecturas visuales, resolviéndose las discrepancias a través de una tercera persona. En la tira reactiva, se evaluaron los siguientes parámetros: glucosa (reacción cromógena glucosa oxidasa-peroxidasa; rango de medición: 50–1000 mg/dL; 2,8–55,6 mmol/L), y bilirrubina (reacción colorimétrica de sal de diazonio; rango de medición: 0,5–6,0 mg/dL; 8,55–102,6 μmol/L). Lamentablemente, no pudimos analizar las proteínas totales (error proteico de indicadores de pH, rango de medición: 10–1000 mg/dL; 0,1–10 g/L), ni la creatinina (reacción de Jaffe, rango de medición: 10–300 mg/dL; 884,2–26,526 μmol/L), debido a que la tira reactiva tenía un rango de medición demasiado bajo o demasiado elevado, respectivamente.

Los resultados de las pruebas se expresan como medianas y rangos intercuartílicos (RIC). Según la prueba U de Mann–Whitney y el coeficiente de correlación de Spearman, los resultados semicuantitativos de uroanálisis obtenidos con la tira reactiva mostraron una correlación directa con los resultados en muestras pareadas obtenidos en el analizador Roche Cobas 8000. También estimamos la concordancia entre las distintas categorías de concentraciones de glucosa y bilirrubina en plasma, analizadas con las dos técnicas: glucosa 0–4,1; 4,2–8,1; 8,2–13,9, >13,9 mmol/L; bilirrubina: 0–17,0; 17,1–51,3; >51,3 μmol/L. El análisis estadístico se realizó con el programa Analyse-it (Analyse-it Software Ltd, Leeds, Reino Unido). El estudio se realizó en muestras de pacientes sobrantes anonimizadas una vez finalizadas las pruebas ordinarias, por lo que no fue preciso obtener un consentimiento informado.

El estudio se realizó de acuerdo con los principios de la Declaración de Helsinki y de conformidad con la legislación local. Así mismo, el estudio fue aprobado por el Comité Ético del Hospital Universitario de Verona (970CESC, 20 de julio de 2016).

## Resultados

**Figura 1:**
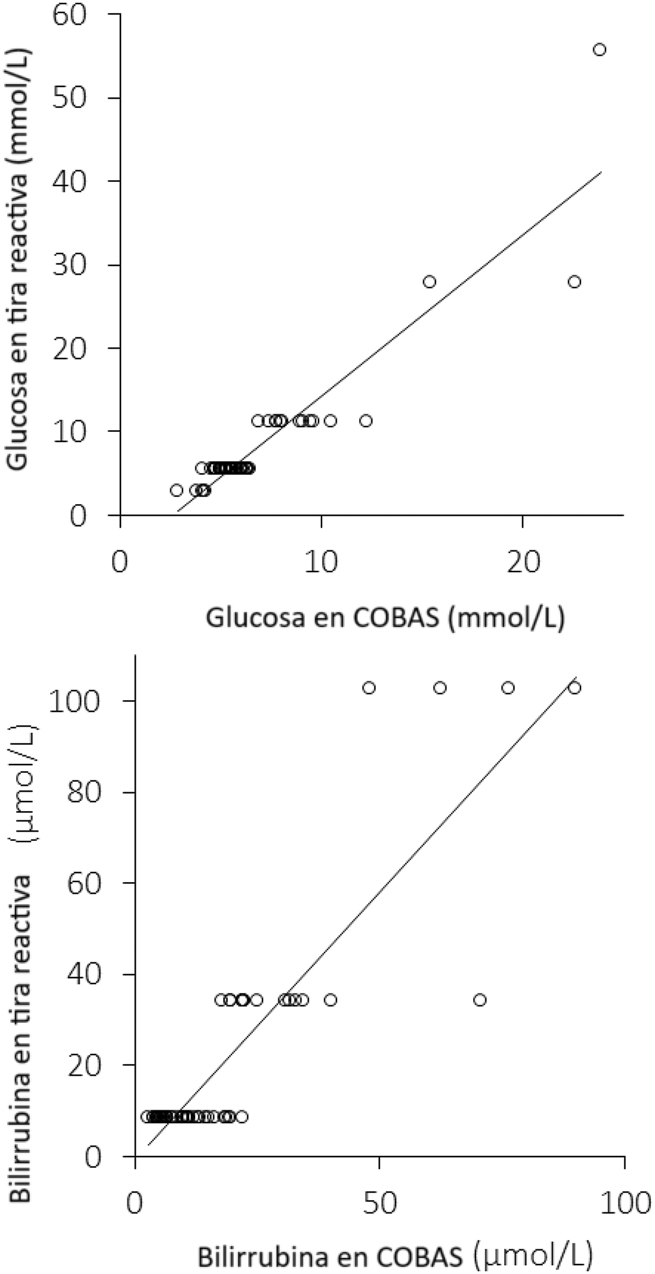
Correlación de Spearman de los resultados de glucosa y bilirrubina en plasma entre Roche COBAS 8000 y la lectura manual de tiras reactivas comerciales para orina.

Los principales resultados del estudio se muestran en la Tabla 1 y la Figura 1. Se observó una diferencia marginalmente significativa en los valores de glucosa en plasma entre COBAS (5,8 mmol/L; IQR, 5,0–6,6 mmol/L) y la tira reactiva (5,6 mmol/L; IQR, 5,6–6,9 mmol/L; p=0,040). Por contra, no se observó ninguna diferencia significativa en la mediana de los valores de bilirrubina en plasma entre COBAS (11,2 μmol/L; IQR, 6,6–22,0 μmol/L) y la tira reactiva (8,6 μmol/L; IQR, 8,6–34,2 μmol/L; p=0,090), respectivamente. El coeficiente de correlación de Spearman entre COBAS y la tira reactiva fue de 0,83 (IC 95 %, 0,73–0,90; p<0,001) para la glucosa en plasma, y 0,78 (IC 95 %, 0,66–0,87; p<0,001) para la bilirrubina en plasma. La concordancia acumulada entre COBAS y la tira reactiva fue alta tanto para la glucosa (88 %; coeficiente kappa, 0,75; IC 95 %, 0,58–0,92; p<0,001) como para la bilirrubina (88 %; coeficiente kappa, 0,76; IC 95 %, 0,60–0.92; p<0,001) (Tabla 2).

**Tabla 1:** Comparación de los resultados de la prueba de glucosa y bilirrubina en plasma entre Roche COBAS 8000 y la lectura manual de la tira reactiva comercial para orina.

Parámetro	Roche COBAS 8000	Tira reactiva	Valor p
Glucosa, mmol/L	5,8 (IQR, 5,0–6,6)	5,6 (IQR, 5,6–6,9)	0,040
Bilirrubina, µmol/L	11,2 (IQR, 6,6–22,0)	8,6 (IQR, 8,6–34,2)	0,099

**Tabla 2:** Concordancia de los valores de glucosa y bilirrubina en plasma medidos en Roche COBAS 8000 y con una tira reactiva comercial para orina.

Glucosa, mmol/L	Tira reactiva				Total
COBAS	Entre 0 y 4,1	Entre 4,2 y 8,1	Entre 8,2 y 13,9	>13,9	
Entre 0 y 4,1	4	1	0	0	5
Entre 4,2 y 8,1	1	39	5	0	45
Entre 8,2 y 13,8	0	0	7	0	7
>13,9	0	0	0	3	3
Total	5	40	12	3	60

Bilirrubina, µmol/L	Tira reactiva			Total
COBAS	Entre 0 y 17,0	Entre 17,1 y 51,3	>51,3	
Entre 0 y 17,0	37	0	0	37
Entre 17,1 y 51,2	5	13	1	19
>51,3	0	1	3	4
Total	42	14	4	60

## Discusión

En entornos con recursos limitados, así como en áreas afectadas por catástrofes como guerras, terremotos, tsunamis, inundaciones, incendios forestales, etc. [6], el acceso a pruebas diagnósticas puede verse dificultado por la falta de recursos, herramientas y/o infraestructuras diagnósticas, o la ausencia de profesionales. Aunque en estos contextos se pueden realizar esfuerzos para mejorar o recuperar el acceso a pruebas diagnósticas, las dificultades pueden ser insalvables [6]. La disponibilidad de pruebas manuales, rápidas y poco costosas que proporcionen el resultado (aunque sea semicuantitativo) de algunas pruebas analíticas esenciales, se postula como una opción más que deseable. Para tal fin, diseñamos este estudio de prueba de concepto, con el fin de investigar si las tiras reactivas, que se pueden interpretar visualmente por profesionales sanitarios o por los propios pacientes, podrían sustituir temporalmente a otras pruebas de laboratorio más exactas, ofreciendo así algunos parámetros básicos, que podrían dar una orientación diagnóstica y facilitar la toma de decisiones médicas.

Los resultados del presente estudio de prueba de concepto indican que las tiras reactivas comerciales para orina que empleamos poseen un rendimiento aceptable a la hora de determinar las concentraciones de glucosa y bilirrubina en plasma, en comparación a las pruebas analíticas de referencia. Limitamos el estudio a estos dos parámetros, ya que los rangos de los demás parámetros incluidos en la tira reactiva no son contrastables en plasma y orina. De este modo, en aquellos casos donde sea extremadamente urgente o necesario obtener información adicional sobre el estado de un paciente, pero no se disponga de analizadores de laboratorio, se podría utilizar de manera puntual la tira reactiva para orina para evaluar las concentraciones de glucosa y bilirrubina, si se cuenta con medios de separación de muestras (por ejemplo, pequeñas centrifugadoras manuales, relativamente baratas, que se pueden adquirir por unos 100 euros). Estos resultados, también podrían abrir la puerta a desarrollar tiras diagnósticas específicas para plasma, suero, o incluso sangre total, que se pudieran utilizar en lugares donde, de forma puntual o permanente y, por diversas razones, no se pudieran utilizar las pruebas analíticas convencionales (ya sea con analizadores de laboratorio tradicionales o con dispositivos de punto de atención). Es innegable que estos mismos estudios se pueden realizar con multitud de pruebas de punto de atención (POCT). Sin embargo, estas tienen un coste, y aunque la tira reactiva tenga un precio similar, el coste adicional de la instrumentación que precisan las POCT superaría el de la tira reactiva manual.

El presente estudio de prueba de concepto demuestra que las tiras reactivas son más adecuadas en escenarios en los que no se disponga de equipamiento de laboratorio y/o este no se pueda adquirir por razones económicas.
